# Supplementary material for: Pore Formation Mechanism of A-Beta Peptide on the Fluid Membrane: A Combined Coarse-Grained and All-Atomic Model
Source: Molecules. 2022 Jun 18;27(12):3924. doi: 10.3390/molecules27123924 (PMC9231318; doi:10.3390/molecules27123924)
Supplement: Supplementary file 1 [file molecules-27-03924-s001.zip › molecules-1736728-supplementary.pdf]

Supporting information

Pore formation mechanism of A-beta peptide on the fluid membrane: A combined coarse-grained and all-atomic models

Yuxi Dai [1], Zhexing Xie [2] and Lijun Liang [1,*]

[a.] College of Automation, Hangzhou Dianzi University, Hangzhou 310018, People's Republic of China

[b.] College of Accounting, Hangzhou Dianzi University, Hangzhou, 310018, China

* Correspondence: llj@hdu.edu.cn

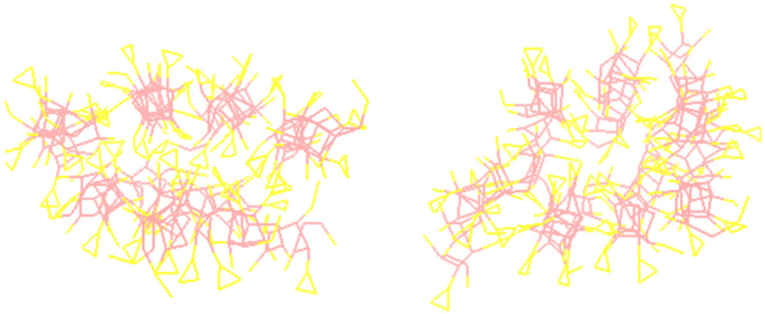

A. Fiber-like formation B. Pore-like formation

Figure S1. The structure of Aβ peptides aggregation: (**A**) fiber-like formation and (**B**) pore-like formation.
